# Biologging, Remotely-Sensed Oceanography and the Continuous Plankton Recorder Reveal the Environmental Determinants of a Seabird Wintering Hotspot

**DOI:** 10.1371/journal.pone.0041194

**Published:** 2012-07-18

**Authors:** Jérôme Fort, Grégory Beaugrand, David Grémillet, Richard A. Phillips

**Affiliations:** 1 Department of Bioscience, Aarhus University, Roskilde, Denmark; 2 Laboratoire d’Océanologie et de Géosciences, UMR 8087 du CNRS, Station Marine, Université des Sciences et Technologies de Lille 1, Wimereux, France; 3 Sir Alister Hardy Foundation for Ocean Science, Plymouth, United Kingdom; 4 Centre d’Ecologie Fonctionnelle et Evolutive, UMR 5175 du CNRS, Montpellier, France; 5 Percy FitzPatrick Institute, DST/NRF Centre of Excellence, University of Cape Town, Rondebosch, South Africa; 6 British Antarctic Survey, Natural Environment Research Council, Cambridge, United Kingdom; Hawaii Pacific University, United States of America

## Abstract

Marine environments are greatly affected by climate change, and understanding how this perturbation affects marine vertebrates is a major issue. In this context, it is essential to identify the environmental drivers of animal distribution. Here, we focused on the little auk (*Alle alle*), one of the world’s most numerous seabirds and a major component in Arctic food webs. Using a multidisciplinary approach, we show how little auks adopt specific migratory strategies and balance environmental constraints to optimize their energy budgets. Miniature electronic loggers indicate that after breeding, birds from East Greenland migrate >2000 km to overwinter in a restricted area off Newfoundland. Synoptic data available from the Continuous Plankton Recorder (CPR) indicate that this region harbours some of the highest densities of the copepod *Calanus finmarchicus* found in the North Atlantic during winter. Examination of large-scale climatic and oceanographic data suggests that little auks favour patches of high copepod abundance in areas where air temperature ranges from 0°C to 5°C. These results greatly advance our understanding of animal responses to extreme environmental constraints, and highlight that information on habitat preference is key to identifying critical areas for marine conservation.

## Introduction

Improvements in biotelemetry and satellite remote-sensing have led to a substantial increase over the last decade in studies of animal distribution, including in remote marine and polar regions (e.g. [1], [2]). These investigations have greatly improved our knowledge of seasonal movements and the location of biodiversity hotspots, the protection of which is essential for the conservation of multiple species [3]. Within the current context of climate change and rapid habitat modification, understanding the environmental drivers of animal movements and distribution is crucial for predicting their capacity to respond to changing conditions, and ultimately, the consequences for population dynamics [4]. For instance, winter storms, which kill thousands of seabirds every year in the North Atlantic because they disrupt feeding conditions and reduce foraging efficiency, are forecasted to increase in frequency and intensity with climate warming [5], and might therefore have a major impact on populations of wintering seabirds. Similarly, the predicted increase of sea surface temperatures may upset the energy balance of wintering seabirds [6], or the profitability of particular foraging areas [7]. Hence, in order to predict potential future consequences of increasingly severe winter storms and of other expected modifications in the marine environment [8], it is now essential to greatly improve our knowledge of seabird habitat use during winter, and to understand the role of environmental conditions in determining their movements and distribution.

Among North Atlantic seabirds, the little auk (*Alle alle*) holds an important place. Little auks feed on zooplankton (mainly *Calanus* copepods), and to satisfy their daily energy demand must catch tens of thousands of individuals per day at depths on average of 12 m, and to a maximum of 50 m [9]. Given this high consumption, an estimated population of 80 million individuals, and a wide distribution ranging from Canadian to Russian coasts [10], this tiny species of ∼150 g represents a key component of North Atlantic and Arctic marine food webs. During winter, little auks face severe energy constraints [6], but survive in icy North Atlantic waters [11]. Although a waterproof insulating plumage is a considerable advantage, heat loss by little auks in cold air and water is extremely high during this period, and their field metabolic rate per gram body tissue is for instance ten times greater than in an emperor penguin *Aptenodytes forsteri* [6], [12]. Nevertheless, our comprehension of the processes shaping their winter distribution and ecology, particularly their strategies for surviving the extreme conditions, remains poor.

Using a multidisciplinary approach combining biologging, at-sea zooplankton sampling and satellite oceanography, the aims of our study were to determine the winter distribution of little auks breeding in East Greenland and to investigate the key environmental factors governing their migration strategies and habitat use.

## Materials and Methods

### (a) Seabird Winter Distribution

Using miniaturized light-level archival tags (Global Location Sensors, GLS [13]) deployed between 2009 and 2010, we investigated the winter (December-January) distribution of 36 little auks breeding at Kap Höegh (70°44′N, 21°35′W). This colony, where more than 3.5 million pairs of little auks are estimated to be breeding [14], is located close to Scoresby Sund, East Greenland, the second most important breeding site in the World for little auks after the Thule District in Northwest Greenland [15]. During summer 2009, eighty eight breeding little auks were gently caught by hand in their nest crevices. Each bird was then weighed, equipped with a GLS (Mk14, British Antarctic Survey; mass  = 1.4 g, ∼1% of adult body mass) mounted on a conventional metal leg ring with cable ties, and released into its nest chamber after less than 10 minutes of handling. During summer 2010, 50 equipped birds were resighted (57%), and 47 were recaptured (either by hand in their nest or using noose carpets when the bird was standing on rocks), and the logger retrieved (53%). All handling was performed in the shade to avoid heat stress and the head of the bird was covered throughout in order to minimize stress. The resighting rate observed for GLS-equipped little auks was similar to that of a control group of birds captured and recaptured in the same way in 2009 and 2010, respectively, and only marked with a metal ring (61%, Fisher exact test, p = 0.83). Moreover, bird body mass was compared between the two years, and this showed no effect of the logger on body condition (*t*-test: *t* = 0.9, df = 68, p = 0.37). Light data were extracted, analysed and converted into positions using TransEdit and BirdTracker (British Antarctic Survey), following [16] (using threshold of 16, angle of elevation of −4,0°, and applying the compensation for movements). For each equipped bird, two positions were obtained per day (at local noon and midnight). Since these positions were of high quality, no additional filtering process was applied. Little auks are located offshore during the entire study period and the number of positions was therefore very similar for each individual. Bird distribution during the winter was calculated across the North Atlantic as the number of individual positions recorded during December and January in each grid cell of 1°×1°. This method allows an analysis of the relationship between relative bird density and environmental characteristics (copepod distribution, air and water temperatures; see below) within each cell. In order to avoid any bias in the analyses relating to breeding status, only data from birds that bred in summer 2010 were included (n = 36).

### (b) Calanus Copepod Winter Distribution

We estimated the winter distribution and abundance of *Calanus* spp. throughout the North Atlantic. To this end, we used zooplankton data originating from the Continuous Plankton Recorder (CPR) survey.

The CPR survey is an upper-layer plankton monitoring programme that has regularly collected samples in the North Atlantic and adjacent seas at monthly intervals since 1946 [17]. We focussed our analysis on three *Calanus* species (*C. finmarchicus*, *C. glacialis*, *C. hyperboreus*), which are important prey for little auks [11]. Since larger copepodite stages tend to be the preferred prey of *Calanus* predators, including little auks [18], we present results for large copepodite (CV and CVI) stages only. Data were spatially interpolated using the inverse squared distance interpolation procedure [19] for every month and each 2-hour period, integrating 50 years (1958–2007) of data. Therefore, a total of 144 maps were produced for each *Calanus* species. The annual maximum was calculated for each grid cell of 1°×1° and each map depicts the value for a particular location and time of year, expressed as a percentage of this annual maximum. For comparison with the bird tracking data, estimations presented hereafter were calculated for the core-winter months (December-January) and daylight time only (period during which little auks concentrate their foraging effort [9]). During the 1958–2007 period, the spatial distribution of *Calanus* has remained stable in the northwest Atlantic [7] allowing data to be combined for comparison with little auk distribution in 2009/2010.

### (c) Climatic and Oceanographic Conditions

Long-term monthly air temperature and Sea Surface Temperature (SST) data for the period 1960–2005 were mapped throughout the North Atlantic in order to compare little auk distribution with environmental characteristics. These latter data were obtained from the COADS 1-degree enhanced dataset provided by the comprehensive NOAA-CIRES Climate Diagnostics Center Database (Boulder, Colorado, USA).

### (d) Estimation of the Environmental Determinants of Little Auk Winter Distribution

We estimated the wintering niche of little auks as a function of two important variables: temperature (air and sea surface temperature) and the abundance of *Calanus* during the daytime (12∶00–14∶00 period). To characterise the niche, we estimated the number of little auks in December 2009 and January 2010 for each category of air temperatures ranging from −10°C to 19 using intervals of 0.5°C and each category of *C. finmarchicus*, *C. glacialis* and *C. hyperboreus* ranging from 0 to 3.5 (abundance as mean number of individuals per CPR sample, expressed in log_10_(x+1)) using intervals of 0.25. The same analysis was performed with SST ranging from −2°C to 19°C using intervals of 0.5°C.

To examine the relationship between the abundance of little auk and the abundance of *Calanus*, we averaged the number of little auk per category of *Calanus*. To examine the relationship between the abundance of little auk and temperature, we averaged the number of little auk per category of temperatures (air temperature and SST).

For both December and January, the number of grid cells considered in the analysis was 3694 for air temperature, 4732 for little auks, 2614 for *C. finmarchicus*. This type of biological matrix contains many zeros, which have a clear meaning. The high proportion of zeros did not affect our results because relationships were determined in the Euclidean space of the niche. To account for spatial autocorrelation for relationships investigated in the geographical space (e.g. between air temperature and the copepod abundance), the degrees of freedom were recalculated to indicate the minimum number of degree of freedom (df*) needed to maintain a significant relationship at p = 0.05 [20]–[22]. The smaller the df*, the less likely it is that spatial autocorrelation affects the probability of significance.

We then tested whether the abundance of little auk (as log_10_(x+1)) L was the product of a linear relationship with the abundance of *C. finmarchicus* c (as log_10_(x+1)) and a Gaussian relationship with air temperature t (see results), as follows:
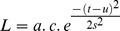



With a the coefficient of proportionality of the linear relationship between the little auk and *C. finmarchicus*, u and s the thermal optimum and thermal tolerance of little auk, respectively. The linear relationship between little auk density and *C. finmarchicus* determines the maximum abundance of little auk at its thermal optimum. The coefficients a, u and s were found by minimising the sum of squares of the residuals assessed by calculating the differences between observed and predicted abundance of little auk (log_10_(x+1)):
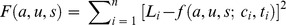



With n the number of observed data.

## Results and Discussion

**Figure 1:**
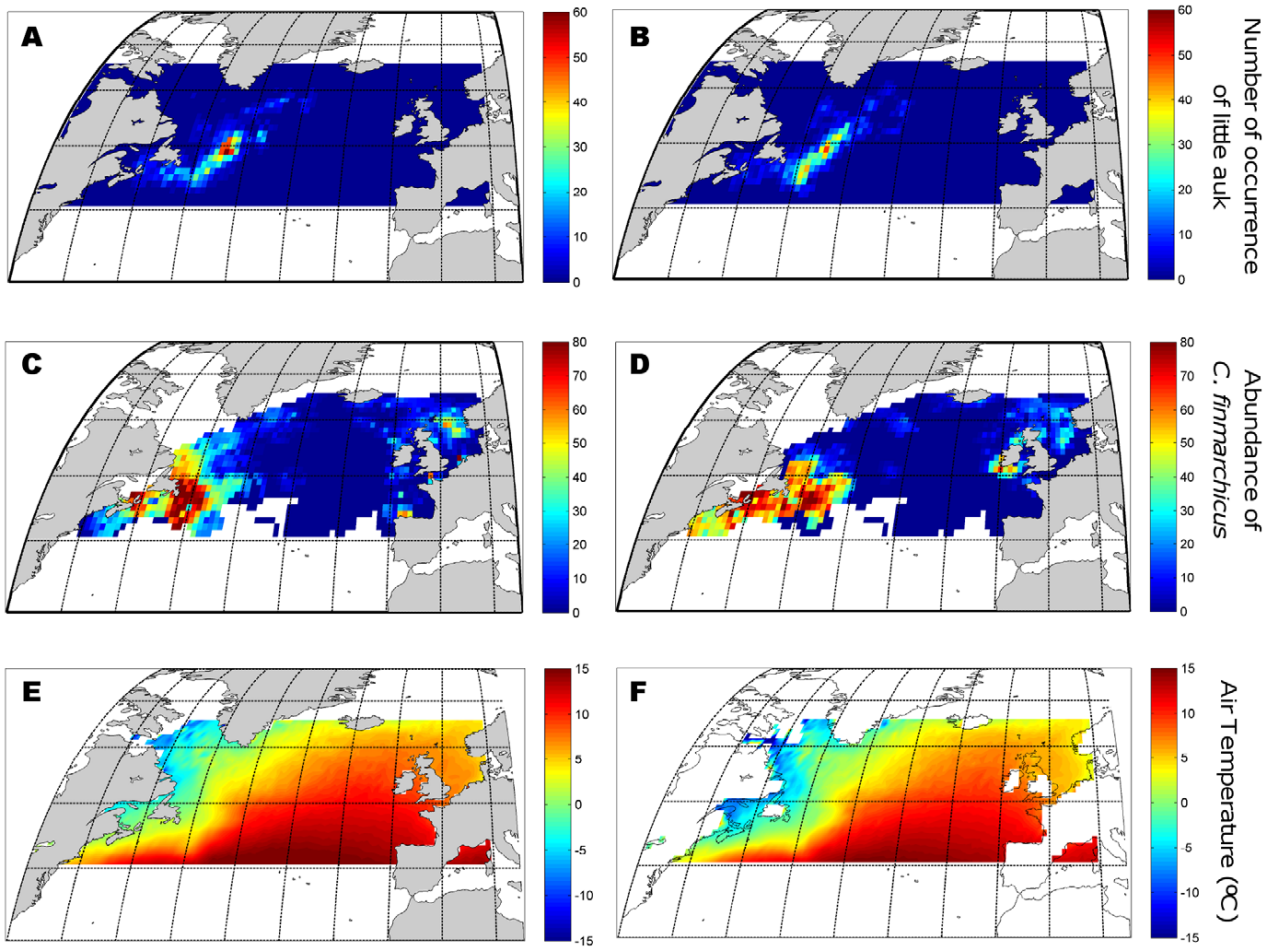
Distribution of little auks, *Calanus finmarchicus*, and air temperature during winter in the North Atlantic Ocean. (A, B) Number of occurrences of little auks per grid cell in December 2009 and January 2010, respectively. (C, D) Abundance of *C. finmarchicus* in December and January, respectively (average for 1958–2007), for the 12∶00–14∶00 period (expressed as percentage of abundance compared to the annual maximum abundance). (E, F) Air temperatures above sea surface (average for 1960–2009) in December and January, respectively.

Little auks, like all seabird species, are free from central-place constraints during winter, and their overall winter range extends over most of the North Atlantic, north of ∼45°N [10]. Despite this wide area of potentially suitable habitat, our analysis shows that all tracked birds migrated ca. 2500 km southwest to a specific area of about 200,000 km^2^ located off Newfoundland (Figures 1A and 1B) that was until recently [23]–[26] not thought to be targeted by wintering seabirds. The aggregated distribution of little auks during December and January (Figures 1A and 1B) strongly suggests they have very specific habitat requirements.

We therefore examined the potential relationship between little auk distribution and several key environmental parameters; the abundance of potential prey, air and Sea Surface Temperature (SST). Several studies suggest that these factors have a major influence on energy balance in seabirds [6], [27], and are important determinants of survival and overwintering strategies in marine endotherms in general [28], [29].

Three calanoid copepod species comprise the main source of food for breeding little auks: *Calanus hyperboreus*, *C. glacialis* and *C. finmarchicus* [11]. During winter, there is very little information on their diet. Previous isotopic investigations suggested that during late winter, little auks from the northwest Atlantic might also feed preferentially on copepods when available within the top 50 m of the water column [9], [30]. A recent investigation shows that euphausiids, *Themisto* spp. and capelin *Mallotus villosus* can also be consumed in coastal areas (Rosing-Asvid unpublished). Using data from the Continuous Plankton Recorder (CPR) survey [17] of the North Atlantic, we estimated the percentage of abundance of the three *Calanus* species present in surface waters (to ca. 6.5 m deep), and therefore available to little auks, compared to the annual maximum abundance in each geographical cell, at a spatial resolution of 1° longitude ×1° latitude. Although the overall abundance of near-surface *C. hyperboreus* and *C. glacialis* was nil or very low during winter compared to the annual maximum (Figures S1 and S2), our results clearly demonstrate that individuals of *C. finmarchicus* remain regionally highly abundant in near-surface waters during winter (Figures 1C and 1D), and that by far the highest abundance was found off Newfoundland, in the northwest Atlantic (Figures 1C and 1D). This important concentration in *Calanus* was unexpected as many studies suggested that *C.* *hyperboreus*, *C. glacialis* and *C. finmarchicus* are only active in the upper water column (<50 m deep) for a few months per year when abundant phytoplankton densities make this environment particularly rich and profitable [31], and that during the autumn, a substantial proportion, mainly copepodite stage V, migrate to depths >500 m, where they remain inactive for several months before returning to the surface in late winter and spring [31]. Hence, *Calanus* availability to near-surface predators such as little auks was thought to be low during winter. Our results contradict this assumption and show that winter diapause behaviour cannot be generalized to *C. finmarchicus* in the northwest Atlantic, as previously suggested by [32].

**Figure 2:**
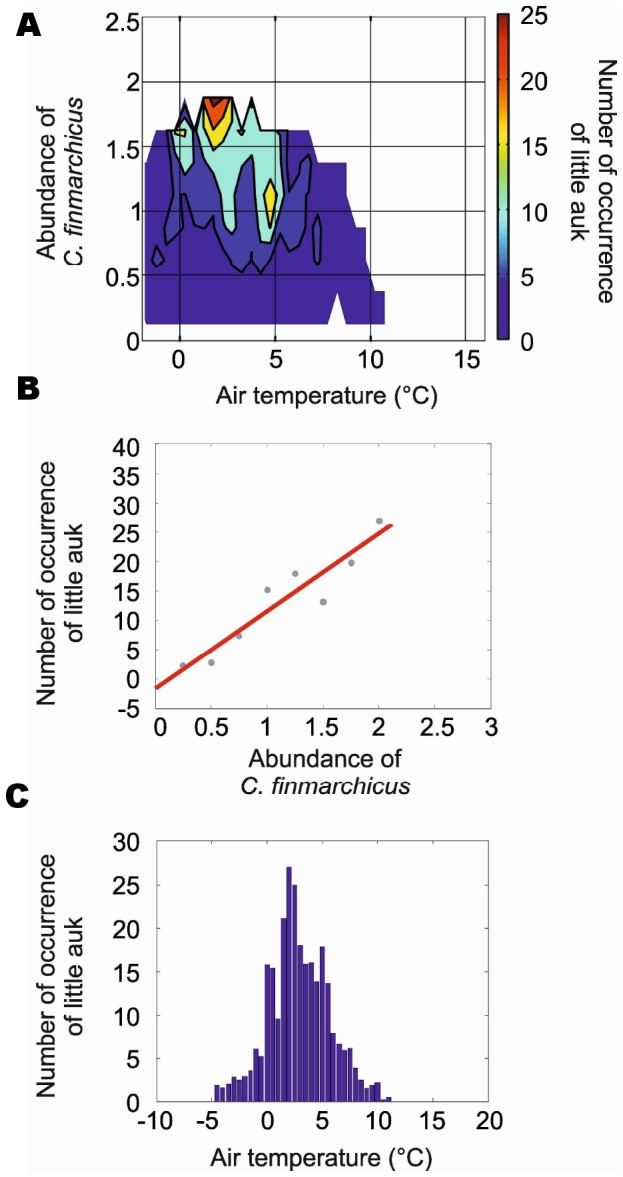
Influence of environmental factors on little auk winter distribution. (A) Occurrence of little auks (December 2009 and January 2010) in relation to *Calanus finmarchicus* densities (12∶00–14∶00 period, average for 1958–2007 – expressed as log10 (x+1)) and to air temperature (average for 1960–2009). (B) Correlation between little auk occurrence (number per grid cell) and abundance of *C. finmarchicus* (12∶00–14∶00 period, average for 1958–2007 – expressed as log10 (x+1)). The data were obtained by reducing the 3D euclidean space in panel a as a 2D euclidean space by averaging the number of little auk occurrence as a function of air temperature. (C) Thermal habitat preference of little auks. The data were obtained by reducing the 3D euclidean space in panel a as a 2D euclidean space by averaging the number of little auk occurrence as a function of *C. finmarchicus* densities.

The highest density of tracked little auks occurred in the eastern part of this region (Figures 1A and 1B), where the winter abundance of *C. finmarchicus* reached up to 80% of the local annual maximum (Figures 1C and 1D). The density of little auks was highly and positively correlated with the the abundance of *C.* *finmarchicus* (Pearson correlation coefficient = 0.94; df = 6; p<0.001; Figure 2B). As both *C. hyperboreus* and *C. glacialis* were absent from most areas where little auks were found, no similar analysis could be conducted for these species. Our results therefore emphasize the importance of an area in the northwest Atlantic for wintering seabirds during a period when plankton abundance is usually considered to be low.

Climatic factors also play a substantial role in determining the overwintering strategy of little auks. Hence, bird occurrence is constrained not only by the abundance and distribution of *C.* *finmarchicus*, but also by air temperature (Figure 2A). Whereas the relationship between little auk density and *C. finmarchicus* is linear and positive (Figure 2B), the relationship with air temperature indicates a clear thermal optimum of about 2°C, (and a narrow thermal range of 0–5°C; Figure 2C). Little auk distribution also showed a relationship with SST, with birds tending to overwinter in areas characterised by optimum SST of 1–7°C (Figure S4). It should be noted that air temperature and copepod abundance also co-varied during winter (r = −0.51, df = 2542, p<0.001). This correlation remains significant at p≤0.05 for df* = 13, which suggest a negligible effect of spatial autocorrelation [20]–[22]. Nevertheless, tracked little auks did not occupy the entire copepod-rich area off Newfoundland but only its eastern extent where temperatures were warmer (Figures 1C–F, Figure 2, Figure S3).

**Figure 3:**
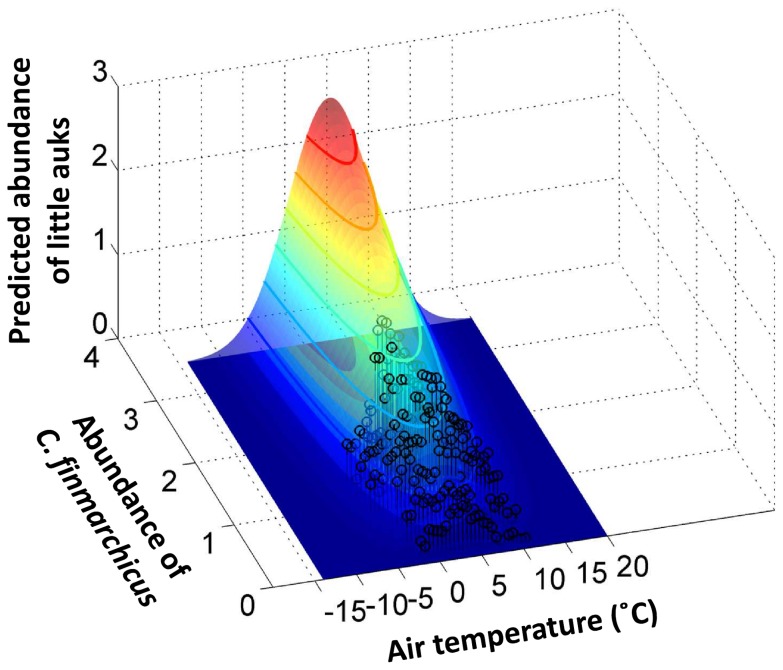
Modelled niche of little auks during winter. Modelled niche of little auks during winter (expressed as log10 (x+1)) as a function of air temperature and abundance of *Calanus finmarchicus* (expressed as log10 (x+1)) for the months of December and January and the 12∶00–14∶00 period (see methods for details). Open circles represent the observed distribution of little auks in relation to air temperature and abundance of *C. finmarchicus* (see Figure 2A).

Variation in air and water temperatures have a high impact on seabird energetics when birds are outside the limits of their thermoneutral zone, resulting in an increase in metabolic rate and thermoregulatory costs (see [33] for little auks and [27] for other diving birds). Through these energy constraints, lower temperatures can then impact the survival of these Arctic seabirds [6]. The avoidance of very cold temperatures by little auks wintering in the northwest Atlantic might therefore keep them within their thermoneutral zone [33], and therefore limit their energy expenditure. Richman and Lovvorn [27] also demonstrated for another alcid species that there was an upper critical temperature above which resting metabolic rate drastically increased. This thermal stress appears when individuals become unable to dissipate body heat [34], and might explain why little auks, a High-Arctic species, avoid warmer temperatures during winter, thereby avoiding the risk of hyperthermia [34]. In this context, further studies on captive little auks would be invaluable for revealing how the energetics of this small seabird varies when outside their thermoneutral zone and the associated physiological consequences. Non-breeding little auks therefore select wintering areas according to different environmental parameters presumably to optimize their energy balance, by maximizing the energy intake while minimizing thermal stress and energy expenditures, in order to promote high winter survival. This is confirmed by our model which explains about 71% of the total variance in the abundance of little auk in the North Atlantic sector for December and January (12∶00–14∶00) (Figure 3).

Using a multidisciplinary approach, our study therefore shows how the environmental conditions encountered by a tiny wintering seabird drive its migratory strategy and its distribution in the North Atlantic. Our results have several important implications. First, different marine top predators, including seabirds such as the little auk, are known to concentrate during winter within hotspots of high resource abundance and biodiversity, which are therefore critical features of marine food webs [35]. Only with the recent increase in tracking studies have some of these areas been identified [23]–[25], and in most cases, the underlying reasons why birds travel there, in terms of their resource requirements and capacity to overcome environmental constraints, are unknown. By showing how seabird distributions reflect strategies to maintain energy balance, and by identifying the key factors involved, we provide a first step toward a much better understanding of their ecological niche, and therefore to ensuring the conservation of an important component of marine food webs. Further studies are now required to determine if little auks breeding in large concentrations in other parts of the Arctic (Northwest Greenland or Svalbard) adopt similar migratory strategies and occupy the same thermal niche as those from East Greenland. Moreover, although our results are in accordance with preliminary investigations for a different year [24], further studies over several years would confirm whether little auks consistently winter in the same area. Such information at a meta-population level could then be incorporated in our model to define, at the species level, the key environmental determinants of their non-breeding movements and distribution. Second, we highlight the existence of a concentrated but extremely rich and profitable resource in the northwest Atlantic, likely to be important for other fish, birds and marine mammals. Recent research indicates that several seabird species target this area during winter, including kittiwakes *Rissa tridactyla*, common *Uria aalge* and Brünnich’s guillemots *U. lomvia* [23], [25], [26]. This important hotspot, extending into Canadian and international waters, might therefore be considered for protection under collaborative management plans. We further hope these findings will stimulate future research on the role of *Calanus* species in North Atlantic food webs during the winter. Third, several studies suggest that ongoing and future climate changes might have important impacts on the distribution, abundance and morphology of *Calanus* species in the North Atlantic. They generally predict a northward movement, with the smaller, low lipid *Calanus* species typical of temperate regions extending their distribution to the north, replacing the larger lipid-rich, cold adapted species [7], [36]. Through these effects and bottom-up control processes [37], and given their dominant role in food webs, environmental change will affect the dynamics of the entire ecosystem. The fate of their many predators, including little auks, will therefore depend on their capacity to adapt, by tracking any shift in the winter distribution of copepods or by switching to new resources within their thermal constraints. Last, beyond the particular relationship between *Calanus* spp. and little auks, we confirm with this multi-species approach the need to better define distribution and interactions between different components of food-webs in order to fully understand the trophodynamics of ecosystems and to predict the impacts of future global change.

## Supporting Information

Figure S1 **Winter abundance of** ***Calanus glacialis*** **in the North Atlantic.** (A, B) abundance of C. glacialis in December and January, respectively (average for 1958–2007), for the 12∶00–14∶00 period (expressed as percentage of abundance compared to the annual maximum abundance).
(TIF)

Figure S2 **Winter abundance of** ***Calanus hyperboreus*** **in the North Atlantic.** (A, B) abundance of C. hyperboreus in December and January, respectively (average for 1958–2007), for the 12∶00–14∶00 period (expressed as percentage of abundance compared to the annual maximum abundance).
(TIF)

Figure S3 **Mean sea surface temperature (SST) in the North Atlantic Ocean.** (A, B) air temperatures above sea surface (average for 1960–2009) in December and January, respectively.
(TIF)

Figure S4 **Influence of SST on little auk winter distribution.** (A) occurrence of little auks (December 2009 and January 2010) in relation to *Calanus finmarchicus* densities (12∶00–14∶00 period, average for 1958–2007 – expressed as log10 (x+1)) and to SST (average for 1960–2009). (B) thermal association preferendum of little auks with SST (average for 1960–2009). The data were obtained by reducing the 3D euclidean space in panel (A) of this figure as a 2D Euclidean space, by averaging the number of little auk occurrence as a function of *C. finmarchicus* densities.
(TIF)
