# Supplementary figures and images for: Biologging, Remotely-Sensed Oceanography and the Continuous Plankton Recorder Reveal the Environmental Determinants of a Seabird Wintering Hotspot
Source: PLoS One. 2012 Jul 18;7(7):e41194. doi: 10.1371/journal.pone.0041194 (PMC3399871; doi:10.1371/journal.pone.0041194)

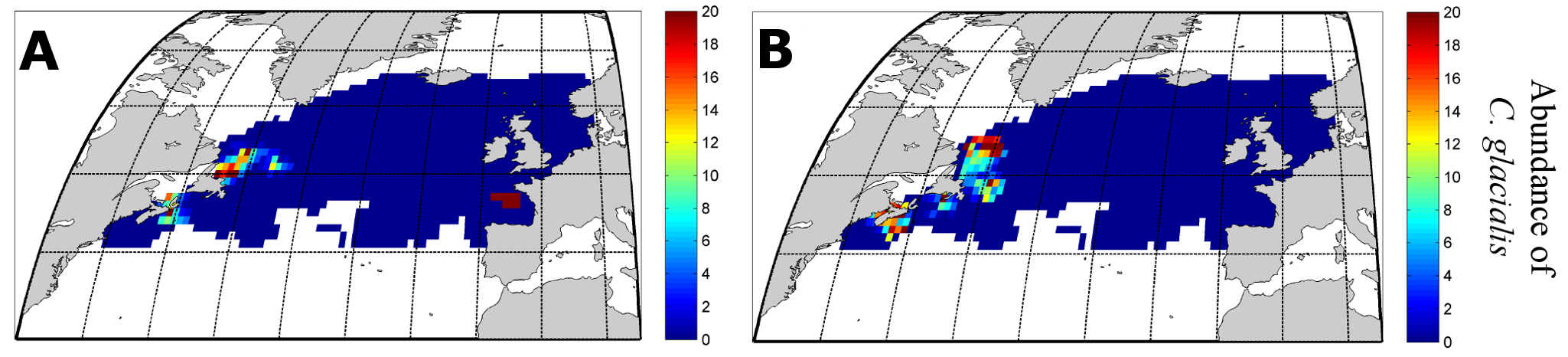

Supplement: Figure S1 — Winter abundance of Calanus glacialis in the North Atlantic. (A, B) abundance of C. glacialis in December and January, respectively (average for 1958–2007), for the 12∶00–14∶00 period (expressed as percentage of abundance compared to the annual maximum abundance). (TIF) [file pone.0041194.s001.tif]

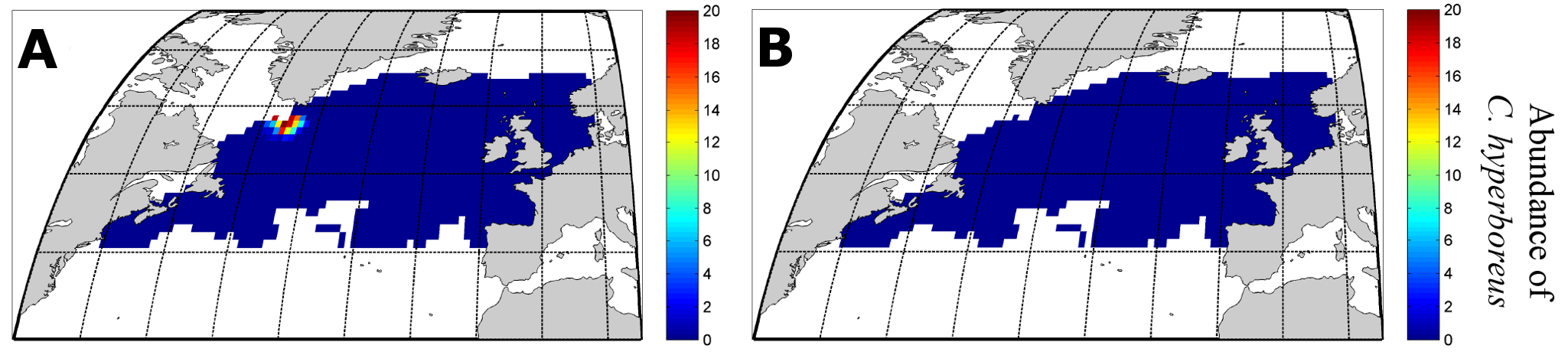

Supplement: Figure S2 — Winter abundance of Calanus hyperboreus in the North Atlantic. (A, B) abundance of C. hyperboreus in December and January, respectively (average for 1958–2007), for the 12∶00–14∶00 period (expressed as percentage of abundance compared to the annual maximum abundance). (TIF) [file pone.0041194.s002.tif]

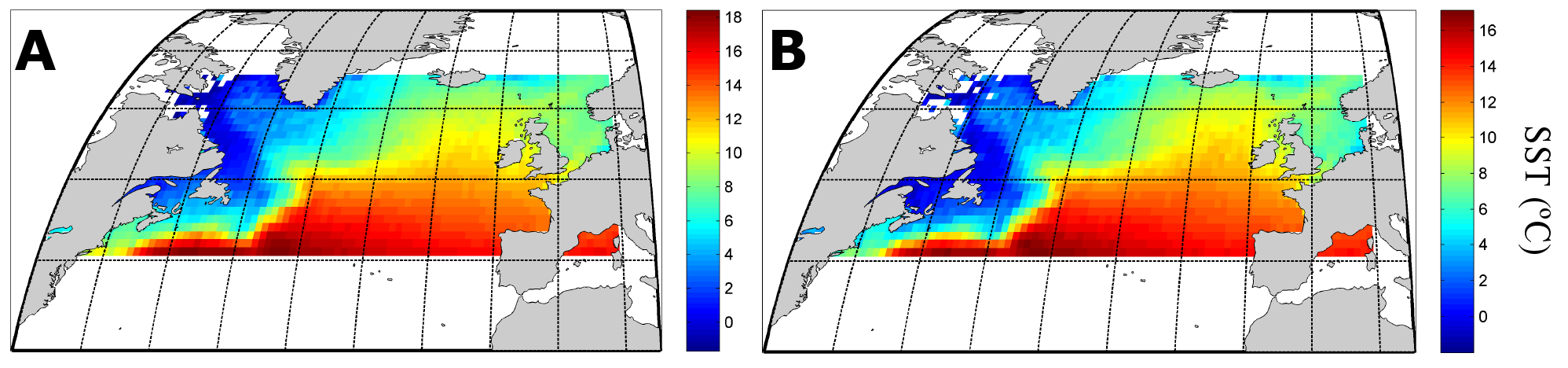

Supplement: Figure S3 — Mean sea surface temperature (SST) in the North Atlantic Ocean. (A, B) air temperatures above sea surface (average for 1960–2009) in December and January, respectively. (TIF) [file pone.0041194.s003.tif]

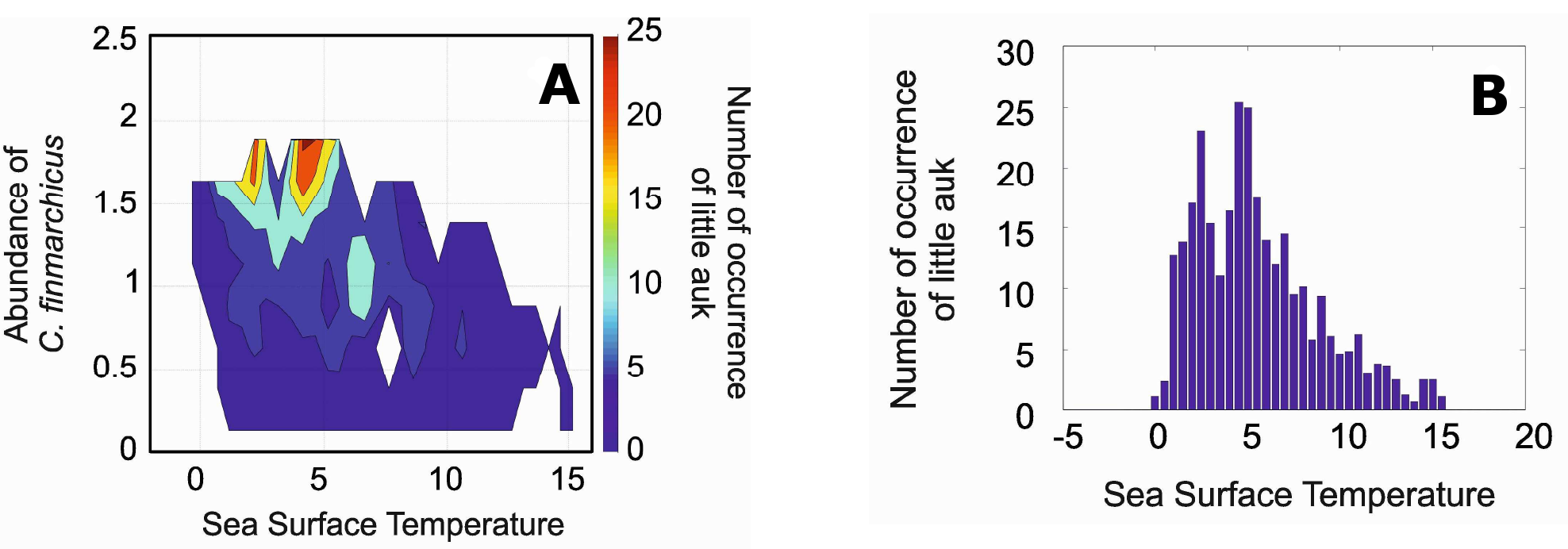

Supplement: Figure S4 — Influence of SST on little auk winter distribution. (A) occurrence of little auks (December 2009 and January 2010) in relation to Calanus finmarchicus densities (12∶00–14∶00 period, average for 1958–2007 – expressed as log10 (x+1)) and to SST (average for 1960–2009). (B) thermal association preferendum of little auks with SST (average for 1960–2009). The data were obtained by reducing the 3D euclidean space in panel (A) of this figure as a 2D Euclidean space, by averaging the number of little auk occurrence as a function of C. finmarchicus densities. (TIF) [file pone.0041194.s004.tif]
